# Simultaneous tibial and fibular sesamoid bone stress injuries in a collegiate volleyball player: The role of shockwave therapy in recovery and return to sport

**DOI:** 10.1002/pmrj.70093

**Published:** 2026-02-09

**Authors:** Bridget M. Doyle, Nader F. Elkabbani, Farah Hussain, Mason W. Briles, Rosa M. Pasculli

**Affiliations:** ^1^ Primary Care Sports Medicine, Stamps Health Services Georgia Institute of Technology Atlanta Georgia USA; ^2^ Division of Sports Medicine, Department of Orthopaedics Emory University School of Medicine Atlanta Georgia USA; ^3^ Department of Radiology and Imaging Sciences Emory University School of Medicine Atlanta Georgia USA; ^4^ Emory Sports Medicine Atlanta Georgia USA

A 21‐year‐old female collegiate volleyball player presented with 4 months of atraumatic left forefoot pain. Her discomfort was initially intermittent and activity related but progressed to persistent pain with ambulation. She denied swelling or bruising. She reported regular withdrawal bleeding while on combined hormonal contraceptives and no history of menstrual irregularity off contraception, with menses starting at age 13. Screening for disordered eating, malabsorption, and prior bone stress injuries (BSIs) was negative.

Examination revealed an antalgic gait and callus formation along the plantar surface near the first metatarsal head, with maximal point tenderness over the medial (tibial) sesamoid and mild tenderness over the lateral (fibular) sesamoid. Her ankle/foot strength and range of motion were normal. The flexor hallucis longus stretch test was negative, and resisted great toe plantarflexion was pain‐free.

Radiographs of the left foot revealed a linear lucency through the distal aspect of the fibular hallux sesamoid, suggesting a nondisplaced fracture, as well as a bipartite tibial hallux sesamoid. Subsequent magnetic resonance imaging confirmed a nondisplaced fracture of the fibular sesamoid with minimal surrounding bone marrow edema, suggestive of an acute to subacute stress fracture. The tibial sesamoid demonstrated bipartite morphology with associated bone marrow edema, slightly more pronounced involving the distal pole. The radiologist noted asymmetric edema across the bipartite segments, suggesting sesamoiditis with possible superimposed BSI (Figure 1). Taken together, these findings support a challenging presentation of simultaneous hallux sesamoid BSIs.

**FIGURE 1:**
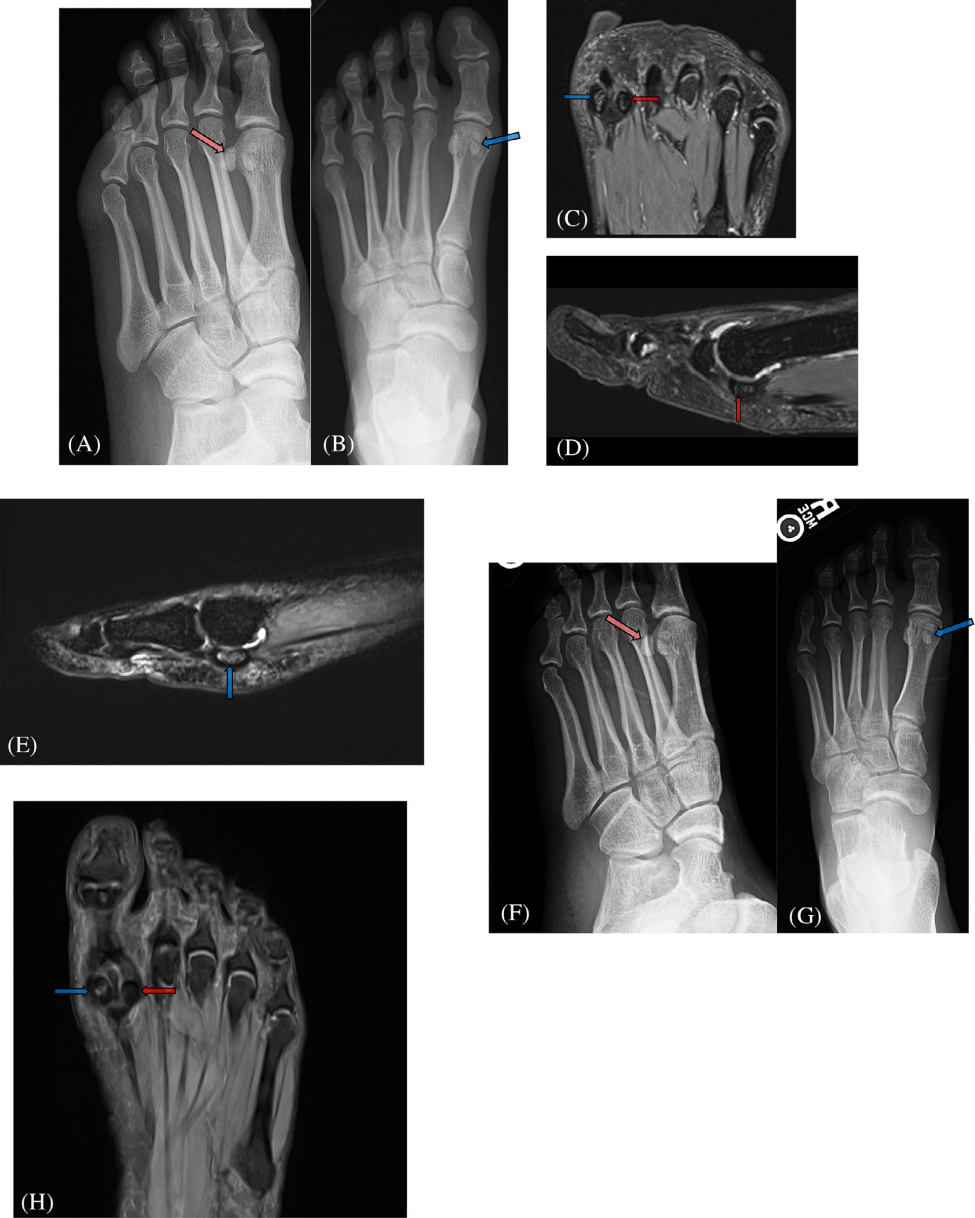
Left foot radiographs (A, B) and magnetic resonance images (C–E). Repeat 1‐year follow‐up left foot radiographs (F) and magnetic resonance images (G–H). (A) Linear lucency through the distal aspect of the fibular hallux sesamoid suspicious for nondisplaced fracture (red arrow). (B). Bipartite tibial hallux sesamoid (blue arrow). (C). Bone marrow edema involving the fibular hallux sesamoid with superimposed fracture line (red arrow). Bipartite tibial hallux sesamoid with associated bone marrow edema (blue arrow). (D). Fracture involving the fibular hallux sesamoid with scant surrounding bone marrow edema (red arrow). (E). Bipartite tibial hallux sesamoid with associated bone marrow edema, more pronounced at the distal pole (blue arrow). (F). Interval resolution of fracture lucency within the fibular hallux sesamoid (red arrow). (G). Bipartite tibial hallux sesamoid (blue arrow). (H). No fracture line or bone marrow edema identified within the fibular hallux sesamoid, consistent with healed stress fracture (red arrow). Interval resolution of bone marrow edema in the bipartite tibial hallux sesamoid (blue arrow).

Initial management included non‐weightbearing in a tall walking boot with the use of a knee scooter for 2 weeks, then she transitioned to weightbearing as tolerated in the boot with a sesamoid pad. She began vitamin D supplementation (2000 IU/day) and had an evaluation with a sports dietitian and psychologist. Comprehensive laboratory work was unrevealing for metabolic risk factors for BSI. There was low suspicion for relative energy deficiency in sport; however, nutrition education was emphasized to promote bone health and recovery.

Despite initial improvement, the patient reported persistent pain during attempts to wean from the boot into a stiff‐soled sneaker at 8 weeks. She underwent three extracorporeal shockwave therapy (ESWT) sessions using an electromagnetic‐focused shockwave device (Duolith, Storz Medical) at an energy‐flux density of 0.4 mJ/mm^2^, frequency of 6 Hz, 2000 pulses per session to both the tibial and fibular sesamoids at maximal points of tenderness.^1^ During ESWT, she successfully transitioned to sneakers with a sesamoid pad gradually over 2 weeks. A rehabilitation program emphasizing progressive forefoot loading and strength restoration was implemented. As her weightbearing status improved, she progressed from stationary bike to elliptical, then to light jogging, and ultimately to running, jump progression, and sport‐specific drills. By week 13, she returned to collegiate competition without symptom recurrence (Figure 2). Subsequent biomechanical assessment identified a hypermobile first metatarsal head with excessive forefoot loading, which was addressed by arch taping with a metatarsal pad and proprioceptive retraining.

**FIGURE 2:**
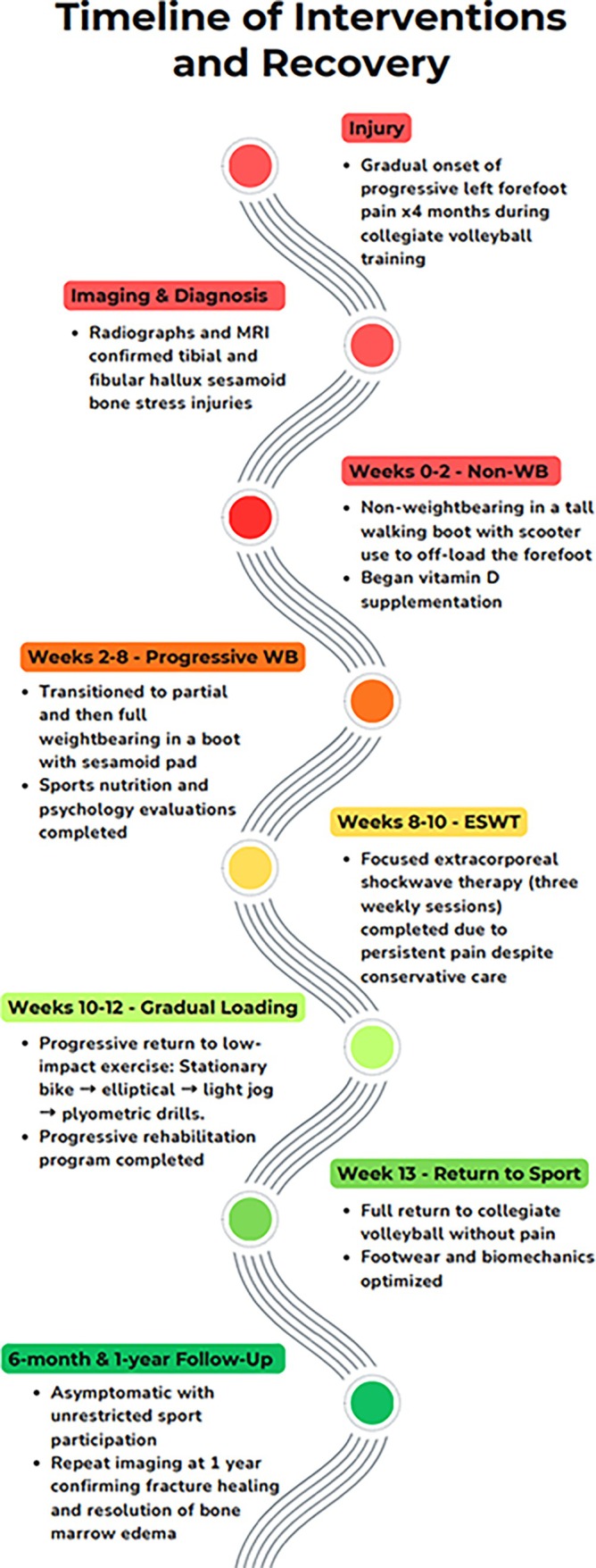
Timeline of clinical course, management, and recovery. Summary of key milestones from injury onset through diagnosis, treatment, and full return to sport in a collegiate volleyball player with simultaneous tibial and fibular sesamoid bone stress injuries. The table outlines each rehabilitation phase, including immobilization, progressive weightbearing, focused extracorporeal shockwave therapy, gradual loading, and follow‐up outcomes through 1 year. Abbreviations: MRI, magnetic resonance imaging; WB, weightbearing; ESWT, extracorporeal shockwave therapy.

Hallux sesamoid BSIs are uncommon but clinically significant injuries, accounting for ∼0.2%–2% of foot and ankle injuries in athletes, and appear to disproportionately affect female athletes.^2^ The tibial sesamoid is more frequently affected than the fibular, and simultaneous involvement of both sesamoids is exceedingly rare, and, to our knowledge, unreported. Although not classified as high risk in the 2025 Delphi Consensus,^3^ hallux sesamoid BSIs may demonstrate delayed union and recurrent symptoms due to limited vascularity and repetitive forefoot loading.

This case emphasizes the importance of individualizing management strategies in athletes with refractory BSIs. Standard conservative care, including activity modification, immobilization, nutrition optimization, biomechanical assessment, and a gradual, structured return to sport, remains the first‐line approach.^4^ When symptoms persist despite appropriate conservative therapy, ESWT can serve as an adjunctive option that promotes angiogenesis and osteogenic activity through mechanotransduction.^5^ Previous studies have demonstrated successful use of focused ESWT for navicular and fifth‐metatarsal BSIs, and more recent reports describe symptom and functional improvement in athletes with chronic hallux sesamoid injuries.^6^, ^7^, ^8^ In this case, clinical improvement followed the ESWT protocol, suggesting a potential role for this modality in refractory sesamoid BSIs.

Although relative energy deficiency in sport was unlikely, the multidisciplinary care team emphasized adequate fueling and recovery strategies. It is important to recognize that even subclinical energy deficiency can impair bone health in athletes. Optimizing nutrition and energy availability is crucial for maintaining bone density, preventing BSIs, and supporting overall athletic performance.^9^

This case provides detailed imaging and therapeutic data for a rare dual sesamoid BSI. Limitations include the inability to exclude contributions from natural recovery and the limited generalizability inherent to a single‐case design. She remained symptom‐free at 6‐month and 1‐year follow‐up evaluations (Figure 2), with repeat imaging confirming fracture healing and resolution of bone marrow edema (Figure 1). Although recurrence risk appears low, continued load management and biomechanical reassessment remain important to prevent reinjury.

This report was prepared in accordance with the CARE Guidelines for case reports, and written informed consent was obtained from the athlete for publication.^10^

## DISCLOSURE

The authors declare no conflict of interest related to this manuscript.

## Data Availability

Data sharing not applicable to this article as no datasets were generated or analysed during the current study.
